# Impacts of geographical locations and sociocultural traits on the Vietnamese entrepreneurship

**DOI:** 10.1186/s40064-016-2850-9

**Published:** 2016-07-27

**Authors:** Quan Hoang Vuong

**Affiliations:** 1 FPT University School of Business, Viet-Uc Building, My Dinh 1, Hanoi, Vietnam; 2 Centre Emile Bernheim, Université Libre de Bruxelles, 50 Ave. F.D. Roosevelt, 1050 Brussels, Belgium

**Keywords:** Entrepreneurship, Creativity, Perseverance, Cultural changes, Transitional economies, L26, M13, O33, P27

## Abstract

This paper presents new results obtained from investigating the data from a 2015 Vietnamese entrepreneurs’ survey, containing 3071 observations. Evidence from the estimations using multinomial logits was found to support relationships between several sociocultural factors and entrepreneurship-related performance or traits. Specifically, those relationships include: (a) Active participation in entrepreneurs’ social networks and reported value of creativity; (b) CSR-willingness and reported entrepreneurs’ perseverance; (c) Transforming of sociocultural values and entrepreneurs’ decisiveness; and, (d) Lessons learned from others’ failures and perceived chance of success. Using geographical locations as the control variate, evaluations of the baseline-category logits models indicate their varying effects on the outcomes when combined with the sociocultural factors that are found to be statistically significant. Empirical probabilities that give further detail about behavioral patterns are provided; and toward the end, the paper offers some conclusions with some striking insights and useful explanations on the Vietnamese entrepreneurship processes.

## Background

Entrepreneurship has been formally recognized in Vietnam since the early 1990s, a few years after the former planned economy had launched its extensive economic reform program. Entrepreneurial efforts by the populace are critically important because they promote creative business ideas, stimulate entrepreneurs to gather resources, hire workers, and transform resources into goods and services for society’s consumption (Frank 1998). One of the hardest parts in learning about entrepreneurship is dealing with sociocultural facets, as these are associated with the elusive nature of preparedness, creativity, perseverance, and the capability of transforming old values into more appropriate ones as the entrepreneurial life starts.

Furthermore, in a country like Vietnam with a long history of more than 4000 years and complicated changes amid waves of geopolitical and socioeconomic changes throughout its history, a frequently omitted factor in studying entrepreneurship is the geographical location. An early work such as Ralston et al. (1999) analyzes sociocultural values in conjunction with geographical differences, but only to a limited extent and with little focus on entrepreneurship. To fill this knowledge gap, this paper uses a data set obtained from a nationwide entrepreneurs’ survey in 2015, taking into account geographical differences, to examine the possible effects of sociocultural traits on Vietnamese entrepreneurship.

The paper starts with a brief literature review discussing major issues that lead to the subsequent consideration of the variables that enter the analytical models. Then it proceeds to a presentation of the analytical models for investigating the research questions. Third, the paper describes the data subsets that correspond to each research question. The fourth section reports the estimation results and associated statistics, and empirical relationships built upon estimated coefficients. The article closes with a concluding section, discussing useful and striking insights.

## A brief literature review

This section discusses a limited body of literature that gives rise to related themes of research and corresponding questions, which this study aims at. In addition, as a data article the discussion that follows in this section does not stand alone, but is attached to a set of new results reported in Vuong et al. (2016a, b), dealing with such important aspects of entrepreneurship as sociocultural traits, networking and creative performance, entrepreneurs’ learning curve, while controlling for the factor of geographical locations. The discussion also leads to relevant variables that will later enter the estimating equations for deeper analysis.

Due to the complex nature and complication arising from a diverse range of entrepreneurial activities and forms, a taxonomy of causes and effects in entrepreneurship processes would hardly be complete and effective, especially when considering the national scale and taking into account sociocultural and geographical factors. Fortunately, building upon the extant literature of entrepreneurship the following select group of factors can be seen as critical to our understanding, which is crucial for further efforts of acquiring new knowledge by examining the survey data reported by this study. Specifically, the select group of factors include: (a) network and creative performance; (b) social responsibilities and entrepreneurs’ perseverance; (c) the transforming of sociocultural values and entrepreneurs’ decisiveness; and, (d) evaluated chance of success in relation to lessons learned from others’ failures.

**Fig. 1:**
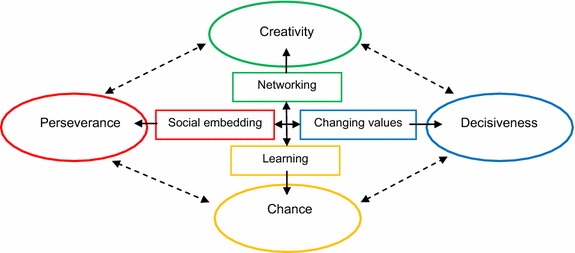
Anchor concepts and flow of logic for analysis

The “anchor concepts” which help identify the key dependent and independent variables that are employed for the data analysis in the study are graphically presented in Fig. 1.

Figure 1 represents two things. First, there are a number of interconnected factors that are either directly or indirectly influential on the others. In this diagram, those groups of factors (in oval shape) are directly influenced by categorical values of causal factors (in rectangular shape), where the solid line and associated arrow indicate the direction of influence. Second, it gives a scope and motivation for estimating effects of factors on some important response outcomes through a conceptual framework based on some extant theories; part of which may be empirically verified by the data set.

### Network and creativity

The first group deals with networking capacity and creativity factors. Creativity is an important part of strategic management research (Runco 2014; Runco and Jaeger 2012) and entrepreneurship studies in both developed and developing economies (Woodman et al. 1993; Ireland et al. 2003; Napier and Vuong 2013) whereas networks represent systems and resources that entrepreneurs need to build up their more sustainable creative performance Vuong et al. (2016a, b).

These factors form what we today consider the “heart” of entrepreneurial attempts, which is expected to induce longer-term competitiveness for entrepreneurial firms, and hence the likelihood of their commercial viability.

Entrepreneurs use social and professional networks to build their capacity and improve their performance, be it skill, experience or creativity capacity (Basadur and Basadur 2013; Basadur et al. 2014). Entrepreneurs’ networking enables the exchange of ideas and information (Runco 1994; Perry-Smith and Shalley 2003), which tend to help improve creativity (Perry-Smith and Shalley 2003; Runco 2014; Vuong and Napier 2014b). It is due to Harryson (2008) that we also learn that “networked innovation” is not only really but powerful as it provides the so-called “strategic navigation” in entrepreneurial settings.

In different economies, and perhaps in different regions of one country, creativity has varying impacts on entrepreneurs’ perceptions about the outcome of their attempts (Frank 1998), which need to be further examined if one wishes to understand entrepreneurship. In Vietnam, there is evidence demonstrating the effect of “destructive creation” whereby an overemphasis on resources is coupled with increasing costs of acquiring resources and a persistent lack of innovation capacity, leading the entrepreneurial firm to financial distress (Vuong and Napier 2014a, b).

### Social responsibilities and entrepreneurs’ perseverance

Due to the social nature of entrepreneurship, social responsibilities (“CSR”) can be considered a naturally embedded value in entrepreneurial processes (Runco 1994; Perry-Smith and Shalley 2003; Runco 2014) and for both creativity and entrepreneurial efforts to produce results, patience is a key ingredient for any success formula (Fillis and Rentschler 2010; Napier et al. 2012; Woodman et al. 1993).

Socially speaking, an entrepreneur has to assemble different types of resources in an entrepreneurial endeavor, and social responsibility is not new (Brush et al. 2008), and closely related to his/her own ambitions (Bosma et al. 2009). In addition, important tasks such as doing the “homework” with an entrepreneurial business plan, forming the team, defining sustained entrepreneurial growth, and so on… all require enormous patience (Vyakarnam et al. 1999; Davidsson 2006; Vuong and Napier 2015). A long-standing learning process that awaits every entrepreneur tends to stress the importance of these factors (Brown 1993; Wagner 2007). As for entrepreneurs, family and relatives, friends and social network peers serve to be their resources in a broad sense (Chang et al. 2009; Zahra et al. 2008); any new venture would need the legitimacy that those social relationships can potentially offer (Meyer and Rowan 1977; Hannan and Freeman 1984; Nagy et al. 2012) or the financial resources, whether traditional (Brown 1993; Weerawardena and Mort 2006) or unconventional (Mollick 2014; Huang and Knight 2015).

As for these interrelated factors, even though social embedding of entrepreneurship has no direct bearing on corporate social responsibility, an actual transforming of sociocultural values—which is typical for such a transition economy like Vietnam—would also tend to transform this into social responsibilities of SMEs in later phase. For the sake simple language, the term “CSR” used in this refers to such social embedding (but not the CSR being the corporate construct in a normal sense) without causing confusion as this deals solely with entrepreneurship. To make it unambiguous, the social legitimacy of entrepreneurship represents both the right and acceptance of a universal system undertaken by a market force, whereby the sphere of influence and base of resources are sociocultural and viewed as longer-term factors.

### Transforming sociocultural values and decisiveness

As any entrepreneurial venture involves a high degree of randomness, uncertainty, and ambiguity (Fillis and Rentschler 2010) an entrepreneur is highly likely to be subject to a process of transforming his or her own sociocultural values until an entrepreneurial mindset that contains emerging values is formed (Vuong and Napier 2015). In this respect, cultural values and entrepreneurial decisions are closely related, and both linked to the entrepreneur’s personality and cognitive style (Woodman et al. 1993; Ward 2004; Vuong and Napier 2015).

This “mindsponge process” (Vuong and Napier 2015) does not occur without the condition of an entrepreneurial self-efficacy construct consisting of such factors as innovation, marketing, management, risk taking, and financial controls, therefore for a large group of prospective entrepreneurs to reach a start decision, their decisiveness will never be obvious and will depend upon the efficient and effective transformations of relevant sociocultural values (Van de Ven et al. 1984; Chen et al. 1998, 2009; Vuong and Napier 2014a). Both theoretical and empirical studies have shown that these values are hard to establish without work experiences and coping strategies (Jennings and McDougald, 2007; Santos et al. 2013). Moreover, since they are not without cost (Markman et al. 2005), entrepreneurs’ decisiveness will also be neither inexpensive nor time-costless (Westhead et al. 2009; Sullivan-Taylor and Branicki 2011; Schindehutte et al. 2006).

### Chance of success and lessons from others’ failures

Since entrepreneurial experiences are in many cases harsh realities and failures (Bosma et al. 2009; Cope 2011; Vuong and Napier 2014a, b), learning from failures helps improve entrepreneurs’ preparedness and confidence, and thus perceived likelihood of success. The learning process involves the understanding of complication arising from the entrepreneurial process, in the forms of increasing risks and unexpected challenges (Santos et al. 2013; Huang and Knight 2015).

The relation between learned lessons and chance of success is also reflected through improved risk appetite and skills for implementing entrepreneurial plans (Hallak et al. 2011; Audretsch and Link 2012), better appreciation of complexity and time lag to business success (Schoonhoven et al. 1990), and enhanced commitment (Zahra et al. 2008).

This review seeks to identify important factors that may form plausible relationships, helping to: i) learn about the relevance of the factors that enter our subsequent analysis of survey data; ii) explore possible relationships and directions of impacts on determination of entrepreneurial pursuits and chance of success/survival; and, iii) have an idea about which factors should be emphasized in an emerging economy context, while controlling for the differences in geographical locations.

## Research questions and method

The consideration of key factors reviewed in the previous section leads to the following statement of research questions.

### Research questions

The research questions that are stated below are derived from the logical framework for interrelated factors provided in Fig. 1 and the above literature review.

RQ1: Does active participation in entrepreneurs’ social networks influence the reported value of creativity?
RQ2: How are entrepreneurs’ willingness to perform social responsibilities and their reported perseverance related?
RQ3: Does entrepreneurs’ capability of adopting emerging sociocultural values translate into their decisiveness in entrepreneuria pursuit?
RQ4: Do lessons learned from others’ failures tend to improve entrepreneurs’ perceived chance of survival/success?

These examinations are controlled for three values of location: North, Center, and South referring to three major geographical divisions in Vietnam that bring to mind distinct sociocultural and economic traits, as suggested by Vuong et al. (2016a, b).

### Research method

To address the above research questions, using the set of categorical data obtained from the survey (described in “Data” section), the subsequent investigation employs the research framework of baseline-category logits (BCL). The subsection below briefly presents key ideas of the analytical framework and the way effects of measured data that reflect behaviors of predictor variables on response variables are examined. A full account of technical treatments following BCL modeling is provided in Agresti (2002) and an alternative to the BCL for analyzing categorical data is the log-linear model with practical analysis provided in Vuong et al. (2013).

### The BCL analytical framework

This study employs the BCL method to investigate the survey data set and its subsets corresponding to each research question. The framework is to estimate a multivariate generalized linear model (GLM), which has the functional form of:

$${\mathbf{g}}({\varvec{\upmu}}_{i} ) = {\mathbf{X}}_{i} {\varvec{\upbeta}},$$

where, ${\varvec{\upmu}}_{i} = {\text{E(}}{\mathbf{Y}}_{i} )$, corresponding to ${\mathbf{y}}_{i} = (y_{i1} ,y_{i2} , \ldots )^{{\prime }}$; row $h$ of the model matrix ${\mathbf{X}}_{i}$ for observation $i$ contains values of independent (also, predictor) variables for $y_{ih}$.

Following this method, as $\pi_{j} ({\mathbf{x}}) = P(Y = j|{\mathbf{x}})$ represent a fixed setting for independent variables, with $\sum\nolimits_{J} {\pi_{j} ({\mathbf{x}})} = 1$, categorical data are distributed over $J$ categories of $Y$ as either binomial or multinomial with corresponding probabilities $\{ \pi_{1} ({\mathbf{x}}), \ldots ,\pi_{j} ({\mathbf{x}})\}$. Thus, the BCL model aligns each dependent (also, response) variable with a baseline category: ${\text{ln[}}{\varvec{\uppi}}_{j} ({\mathbf{x}})/{\varvec{\uppi}}_{J} ({\mathbf{x}}) ]$, with $j = 1, \ldots ,J - 1$.

As $\ln \, [{\varvec{\uppi}}_{a} ({\mathbf{x}})/{\varvec{\uppi}}_{b} ({\mathbf{x}} ) ]= \ln \, [{\varvec{\uppi}}_{a} ({\mathbf{x}})/{\varvec{\uppi}}_{J} ({\mathbf{x}} ) ]- \ln \, [{\varvec{\uppi}}_{b} ({\mathbf{x}})/{\varvec{\uppi}}_{J} ({\mathbf{x}} ) ]$, the set of empirical probabilities from binomial/multinomial logits $\{ {\varvec{\uppi}}_{j} ({\mathbf{x}})\}$ can be computed from the formula:

$${\varvec{\uppi}}_{j} \left( {\mathbf{x}} \right) = \frac{{{ \exp }\left( {\alpha_{j} + \beta_{j}^{\text{T}} {\mathbf{x}}} \right)}}{{1 + \mathop \sum \nolimits_{h}^{J - 1} { \exp }\left( {\alpha_{h} + \beta_{h}^{\text{T}} {\mathbf{x}}} \right)}}.$$

The response and predictor variables used in the investigating models are multinomial and are of categorical value by survey nature. Their coded names, together with values are given in each data subset tabulated following the corresponding research question. An example of a response variable is “inno” referring to the self-reported degree of entrepreneurial creativity/innovation, which has values of: “much,” “some,” or “none”; and of predictor variable “member” referring to the entrepreneur’s activeness in his/her social networks, having values of: “all,” “some,” or “none.”

The actual analysis that is provided in the section on estimations and results follows the practice employed for the same type of data analysis in Vuong (2015).

## Data

This section describes subsets of data extracted from the survey data set, which has been made publicly available in the data article by Vuong (2016) following a 2015 nationwide survey on entrepreneurial activities in different regions of Vietnam, through entrepreneurs’ meetings organized in five regional economic centers (Hanoi, Ho Chi Minh City, Da Nang, Buon Ma Thuot, Can Tho). Entrepreneurs who were willing to join the survey were given information on the purpose of the questionnaire and on how to complete it by authorized personnel. Answers were collected at the end of each event. Among the estimated number of 50,000 entrepreneurs attending these events, the survey team randomly approached about 10,000 during the survey period, from March to May 2015, and later collected a random data sample containing 3071 observations, representing answers in full or in part. In our subsequent analysis, each data subset requires a specific structure reflected through the corresponding tabulated form, with the number of observations used varying depending upon appropriate treatments for missing data (for partial answers).

### Data for RQ1

The first question to be addressed considers the factor creativity, coded in the model as “inno,” in a broad sense, i.e. both creative performance and technological innovation capacity, since it has been regarded as one of the major sociocultural traits of the entrepreneur community and a driver of survival/success for an entrepreneurial effort. As a response variable, creativity has three categorical values: “much,” “some,” and “none,” which identifies if an entrepreneur reports his innovation capacity to be radical (consuming or targeting >50 % of resources including time, funds and workforce), non-radical (10–50 %) or non-existent (<10 %). (The values are predefined and explained by research data team to respondents; thus the categorical values recorded in the data set are universal and coherent to a large extent.) The purpose is to see if the factor “active participation in entrepreneurs’ social networks” may have shown a significant impact on entrepreneurial creativity, controlling for distinct geographical locations (“place” having values of: “north,” “south,” or “central”).

This question has N = 2976 and the first data subset is given in Table 1. The ratio of Northern, Central, and Southern entrepreneurs who participate in the data set is: 13.5, 31.8, and 54.7 %, respectively.

**Table 1:** Data for RQ1: geographical distribution of responses following activeness in social networks and creative performance capacity of entrepreneurs

“place”	“member”	“inno”		
		“much”	“some”	“none”
“central”	“all”	55	86	82
	“none”	38	165	258
	“some”	33	125	101
“north”	“all”	30	47	18
	“none”	30	68	82
	“some”	14	66	45
“south”	“all”	79	181	93
	“none”	86	342	446
	“some”	55	203	139

In all geographical regions, entrepreneurs appear to have not been confident of their creativity capacity. In general only 14 % report positively about their creativity performance, while 1264 (out of 2976) do not see creativity as a significant factor in their business attempts. In addition, entrepreneurs seem to be less connected than most people think them to be, with more than 62 % having no experience of participating in any social networks of entrepreneurs.

### Data for RQ2

The second data subset deals with entrepreneurs’ perseverance (“tforstart”), having values: “less12” (less than 12 months); “b1224” (from 12 to 24 months); and “g24” (until early signs of success). Apart from geographical locations as described above, the factor “csr” (social embedding/corporate social responsibilities) plays an important role in the modeling, which has a value of: “no” (do not see social responsibility as necessary); “yes.sale” (yes, but only if it helps to improve sales); and “yes.resp” (yes, as standard understanding of CSR). In this investigation, N = 2886. The process of data value explanation and collection is similar to what is shown in the subsection Data for RQ1.

The data show that nearly 73 % of respondents intend to pursue their plan despite obstacles until early signs of success. A large portion of entrepreneurs show a tendency to carry out CSR activities, >62 % (see Table 2).

**Table 2:** Data for RQ2: distribution of respondents following factors “perseverance” and “CSR”; controlling for geographical locations

“place”	“csr”	“tforstart”		
		“b1224”	“g24”	“less12”
“central”	“no”	22	27	2
	“yes.sale”	67	215	32
	“yes.resp”	74	455	24
“north”	“no”	4	5	1
	“yes.sale”	25	52	10
	“yes.resp”	52	226	14
“south”	“no”	21	44	15
	“yes.sale”	134	363	44
	“yes.resp”	176	716	66

### Data for RQ3

The third problem deals with the decisiveness of entrepreneurs in starting their business attempt, coded “startplan.” This factor has distinct values of “running” (currently operating an entrepreneurial firm); “soon” (going to start within 12 months); “cond” (only starting when there are favorable socioeconomic conditions); and, “notstart” (not starting); and it serves as the dependent variables in the analysis. Besides the control variate “place,” another predictor is the “mindsponge process” following Vuong and Napier’s (2015) enlarged notion of acculturation, playing the role of independent variables. Coded as “msponge,” this factor demonstrates the extent to which the mindsponge process activates sociocultural values inducting/ejecting mechanisms among entrepreneurs, and has a value of either “strong” (to a large extent), “some” (to a limited extent), or “negl” (negligible). The proxies being used for “mindsponge” are reported readiness of adjusting to emerging sociocultural values by entrepreneurs (in different regions) and actual changes in thinking and behaviors for extant entrepreneurs who have been undertaking some entrepreneurial endeavors.

In this modeling attempt, the data subset has N = 2851 observations and is provided in Table 3.

**Table 3:** Data for RQ3: distribution of entrepreneurs’ entrepreneurial decisions, following effects of “mindsponge process” outcomes, and controlling for geographical locations

“place”	“msponge”	“startplan”			
		“running”	“soon”	“cond”	“notstart”
“central”	“negl”	10	15	75	39
	“some”	42	82	135	52
	“strong”	81	96	226	45
“north”	“negl”	4	8	17	11
	“some”	9	26	58	10
	“strong”	43	90	109	10
“south”	“negl”	10	17	121	79
	“some”	49	129	280	80
	“strong”	90	178	451	74

A first look at the data set unveils that a large portion of entrepreneurs, nearly 52 %, tends to depend on specific socioeconomic conditions to make their decision on whether to start a business or not. In addition, nearly 10 % report that their sociocultural values have been transformed following their actual entrepreneurial attempts as an outcome of the mindsponge process, following Vuong and Napier’s (2015) model.

### Data for RQ4

This last data subset, while controlling for “place,” looks into such factors as valuable lessons learned from past failures (“failurel”) and self-evaluated likelihood of survival/success (“chance”). The factor “failurel” reflects entrepreneurs’ preparedness prior to their entrepreneurial efforts, through learning lessons from past failures in the community, having values of “much” (carefully studied), “some” (to a limited extent), and “none” (little consideration of others’ failures). The factor “chance” has one of the following values: “high” (seeing high chance of survival/success; >80 %), “med” (50–80 %), and “low” (<50 %).

This subset has N = 2842 observations, with its frequency distribution being provided in Table 4.

**Table 4:** Data for RQ4: distribution of entrepreneurs’ reported chance following learning from failure lessons and controlling for geographical locations

“place”	“failurel”	“chance”		
		“high”	“low”	“med”
“central”	“much”	107	24	87
	“some”	188	90	330
	“none”	25	12	20
“north”	“much”	57	12	38
	“some”	87	32	123
	“none”	8	9	12
“south”	“much”	139	37	185
	“some”	317	145	651
	“none”	26	26	55

A quick observation from Table 4 shows that the majority of entrepreneurs pay attention to failure cases they can access, regardless of their estimated chance of success, with the highest ratio belonging to people coming from the central region of the country who see a higher chance of success. This information is interesting as the central region of Vietnam is considered “the land of the poor” where people have shown the sociocultural tradition of learning and thoughtfulness.

## Estimations and results

### Estimations and results for RQ1

Details of estimations for the research question RQ1 is provided in Table 5, with p values mostly being smaller than 0.01, showing the significant influence of predictor variables on the values of the response variables.

**Table 5:** Reported results from RQ1 estimations

	Intercept	“place”		“member”	
		“north”	“south”	“all”	“some”
	*β*_0_	*β*_1_	*β*_2_	*β*_3_	*β*_4_
logit(much|none)	−1.812*** [−14.484]	0.587** [3.278]	0.187 [1.435]	1.470*** [10.616]	0.594*** [4.074]
logit(some|none)	−0.513*** [−6.258]	0.372** [2.785]	0.270** [3.016]	0.810*** [7.567]	0.643*** [6.727]

Signif. codes: 0 ‘***’ 0.001 ‘**’ 0.01 ‘*’ 0.05 ‘·’ 0.1 ‘-’ 1, z-value in square brackets; baseline category for: “place”: “central”, and, “member”: “none”. Residual deviance: 12.13 on 8 d.f

The single largest coefficient is β_3_ = 1.470 with a highly significant p value (<0.0001), showing that activeness in as many social networks as possible exerts a strong influence on increasing the tendency of activating the creative performance factor in determining the outcome of an entrepreneurial effort.

From Table 5, the following empirical relationships (Eqs. RQ1.1–RQ1.2) are derived to “gauge” the influence of geographical locations and active participation in social networks on creativity capacity of entrepreneurs:

RQ1.1 $$\ln \left( {\frac{{\pi_{\text{much}} }}{{\pi_{\text{none}} }}} \right) = - 1.812 + 0.587{\text{North}} + 0.187{\text{South}} + 1.470{\text{allMem}} + 0.594{\text{someMem}}$$

RQ1.2 $$\ln \left( {\frac{{\pi_{\text{some}} }}{{\pi_{\text{none}} }}} \right) = - 0.513 + 0.372{\text{North}} + 0.270{\text{South}} + 0.810{\text{allMem}} + 0.643{\text{someMem}}$$

An example of computing empirical probability from Eqs. (RQ1.1 to RQ1.2) follows:

$$\pi_{\text{much}} = \frac{{{\text{e}}^{{\left( { - 1.812 + 0.587 + 1.470} \right)}} }}{{1 + {\text{e}}^{{\left( { - 1.812 + 0.587 + 1.470} \right)}} + {\text{e}}^{{\left( { - 0.513 + 0.372 + 0.810} \right)}} }} = 0.302$$

There is a 30.2 % probability that an entrepreneur who is located in the Northern region and actively participating in social networks would see his/her entrepreneurial effort to be able to activate the creative performance capacity in his/her entrepreneurship. Other probabilities are computed the same way, and their distribution is provided in Table 6.

**Table 6:** Empirical probability distributions of entrepreneurs’ reported creativity following their social networks’ membership, controlling for geographical locations

“inno”	“much” (a)			“some” (b)			“none” (c)		
“place”|”member”	“all”	“some”	“none”	“all”	“some”	“none”	“all”	“some”	“none”
“north”	0.302	0.167	0.136	0.462	0.519	0.402	0.236	0.314	0.462
“south”	0.237	0.125	0.099	0.487	0.524	0.396	0.276	0.351	0.505
“central”	0.232	0.122	0.093	0.440	0.468	0.340	0.328	0.410	0.567

**Fig. 2:**
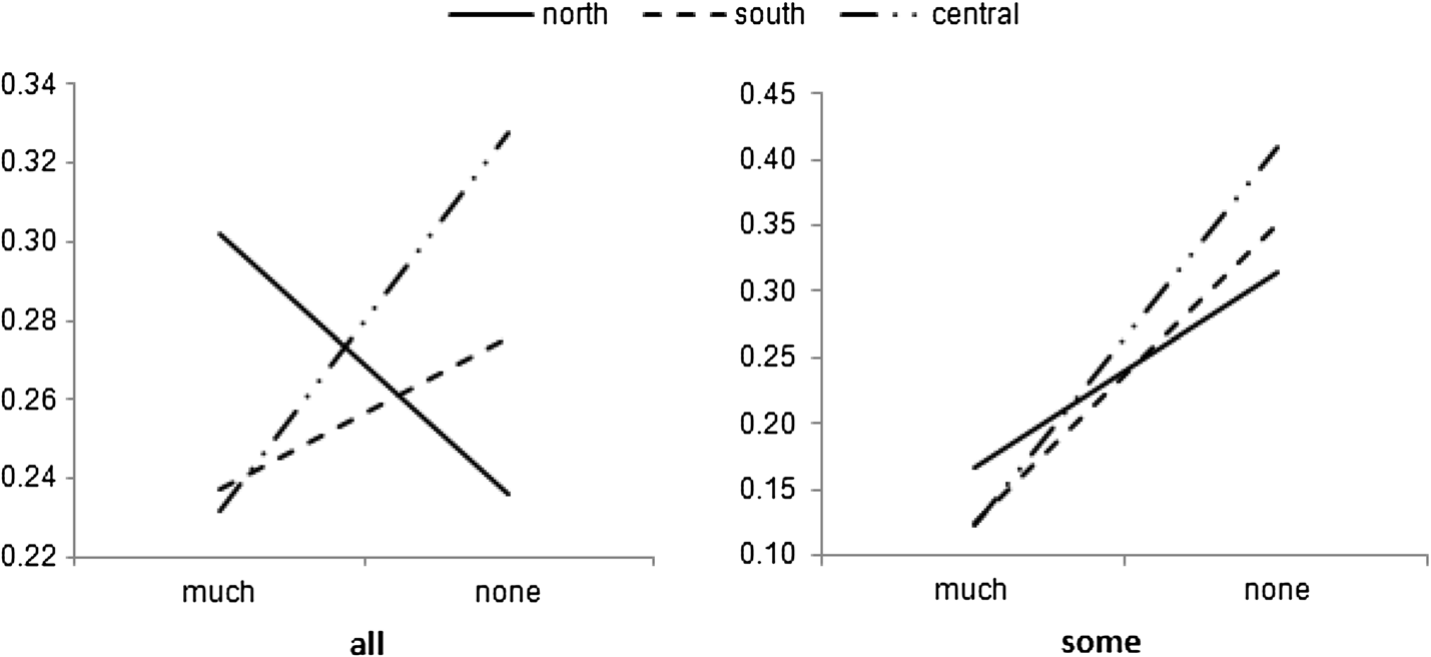
Reported creativity capacity of entrepreneurs coming from different regions, with varying degrees of activeness in social networks

Entrepreneurs coming from regions with different sociocultural traits differ in their perceptions and reliance on their creativity capacity, as shown in Fig. 2, depicting numerical values in Table 6.

### Estimations and results for RQ2

Details of estimations for research question RQ2 is provided in Table 7, with most p values being smaller than 5 %, showing the significant influence of predictor variables.

**Table 7:** Reported results from RQ2 estimations

	Intercept	“place”		“csr”	
		“north”	“south”	“no”	“yes.sale”
	*β*_0_	*β*_1_	*β*_2_	*β*_3_	*β*_4_
logit(b1224|g24)	−1.714*** [−17.313]	0.306* [1.971]	0.243* [2.245]	1.074*** [5.452]	0.519*** [5.145]
logit(less12|g24)	−2.794*** [−17.813]	0.180 [0.713]	0.303^-,·^ [1.819]	1.166*** [4.132]	0.607*** [3.934]

Signif. codes: 0 ‘***’ 0.001 ‘**’ 0.01 ‘*’ 0.05 ‘·’ 0.1 ‘-’ 1, z-value in square brackets; baseline category for: “place”: “central”; and, “csr”: “yes.resp”. Residual deviance: 17.472 on 8 d.f

Generally, the results show that all levels of CSR efforts when combined with geographical locations have significant impacts on defining entrepreneurs’ perseverance in their entrepreneurial efforts. Following the same practice when examining RQ1, Eqs. (RQ2.1–RQ2.2) establish relationships obtained from estimated coefficients of Table 7.

RQ2.1 $$\ln \left( {\frac{{\pi_{{{\text{b}}1224}} }}{{\pi_{{{\text{g}}24}} }}} \right) = - 1.714 + 0.306{\text{North}} + 0.243{\text{South}} + 1.074{\text{noCsr}} + 0.519{\text{yes}}.{\text{saleCsr}}$$

RQ2.2 $$\ln \left( {\frac{{\pi_{{{\text{less}}12}} }}{{\pi_{{{\text{g}}24}} }}} \right) = - 2.794 + 0.180{\text{North}} + 0.303{\text{South}} + 1.166{\text{noCsr}} + 0.607{\text{yes}}.{\text{saleCsr}}$$

Equations (RQ2.1–RQ2.2) then enable the computing of empirical conditional probabilities in Table 8.

**Table 8:** Empirical probability distribution of entrepreneurs’ perseverance following willingness to perform CSR actions, and geographical locations

“tforstart”	“g24” (a)			“b1224” (b)			“less12” (c)		
“place”|”csr”	“no”	“yes.sale”	“yes.resp”	“no”	“yes.sale”	“yes.resp”	“no”	“yes.sale”	“yes.resp”
“north”	0.513	0.647	0.758	0.367	0.266	0.186	0.120	0.087	0.056
“south”	0.516	0.650	0.762	0.347	0.251	0.175	0.137	0.099	0.063
“central”	0.580	0.707	0.806	0.306	0.214	0.145	0.114	0.079	0.049

**Fig. 3:**
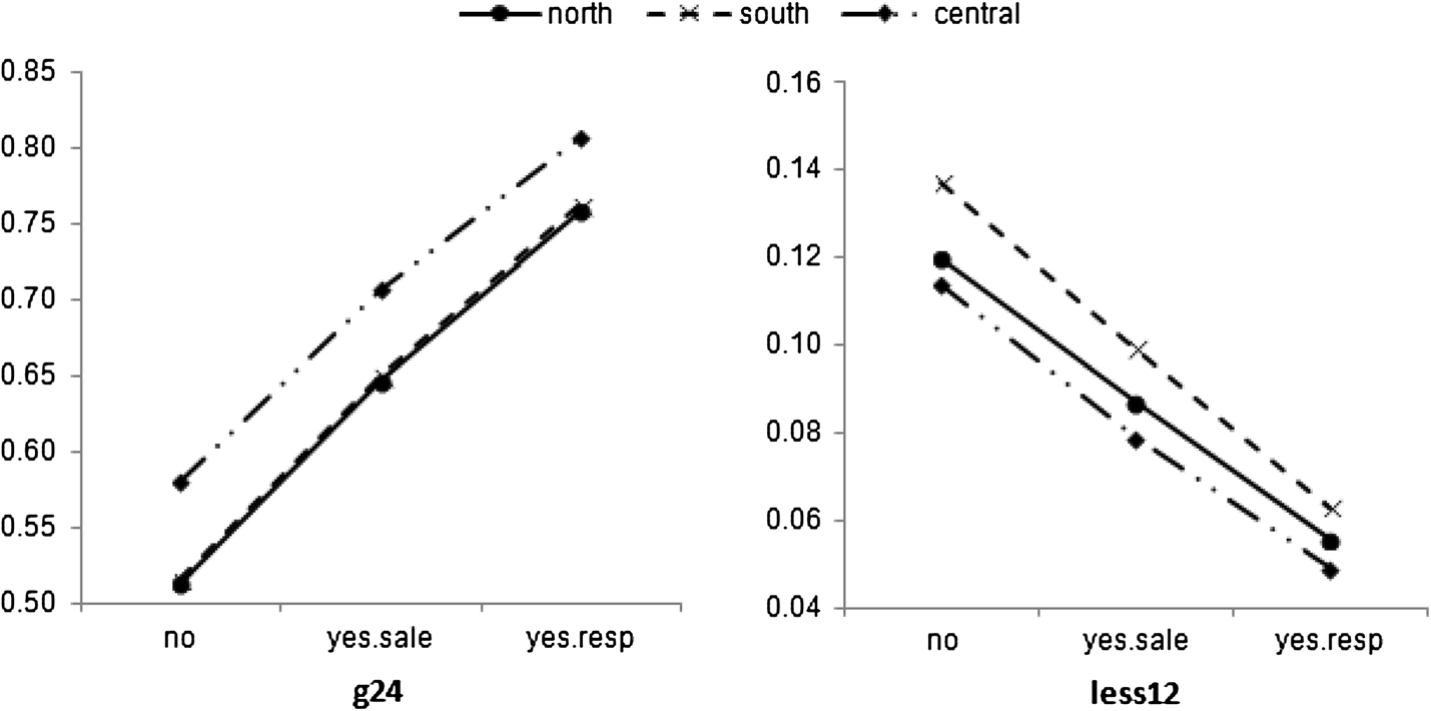
Evidence showing that the sociocultural value of CSR tends to increase the probability among higher perseverance groups

Figure 3 represents a contrast between two entrepreneur groups with different levels of perseverance, controlling for different geographical regions, based on computed numerical values of Table 8.

### Estimations and results for RQ3

Estimating the data of Table 3 helps investigate research question RQ3 on effects of the “mindsponge process” on entrepreneurial decisions. Detailed estimations are provided in Table 9.

**Table 9:** Reported results from RQ3 estimations

	Intercept	“place”		“msponge”	
		“north”	“south”	“negl”	“some”
	*β*_0_	*β*_1_	*β*_2_	*β*_3_	*β*_4_
logit(running|notstart)	0.674*** [4.442]	0.457^-,·^ [1.756]	−0.459** [−2.788]	−2.168*** [−8.688]	−0.824*** [−4.779]
logit(soon|notstart)	0.923*** [6.530]	0.914*** [3.883]	−0.052 [−0.362]	−2.164*** [−10.361]	−0.474** [−3.196]
logit(cond|notstart)	1.682*** [13.259]	0.508* [2.300]	0.108 [0.863]	−1.286*** [−8.753]	−0.584*** [−4.320]

Signif. codes: 0 ‘***’ 0.001 ‘**’ 0.01 ‘*’ 0.05 ‘·’ 0.1 ‘-’ 1, z-value in square brackets; baseline category for: “place”: “central”; and, “msponge”: “strong”. Residual deviance: 17.693 on 12 d.f

Most coefficients are statistically significant with p values being smaller than 0.05. As hypothetical relationships among factors “place,” “msponge,” and “startplan” are accepted, stylized facts are reflected in the Eqs. (RQ3.1–RQ3.3).

RQ3.1 $$\ln \left( {\frac{{\pi_{\text{running}} }}{{\pi_{\text{notstart}} }}} \right) = 0.674 + 0.457{\text{North}} - 0.459{\text{South}} - 2.168{\text{neglMs}} - 0.824{\text{someMs}}$$

RQ3.2 $$\ln \left( {\frac{{\pi_{\text{soon}} }}{{\pi_{\text{notstart}} }}} \right) = 0.923 + 0.914{\text{North}} - 0.052{\text{South}} - 2.164{\text{neglMs}} - 0.474{\text{someMs}}$$

RQ3.3 $$\ln \left( {\frac{{\pi_{\text{cond}} }}{{\pi_{\text{notstart}} }}} \right) = 1.682 + 0.508{\text{North}} + 0.108{\text{South}} - 1.286{\text{neglMs}} - 0.584{\text{someMs}}$$

Then using Eqs. (RQ3.1–RQ3.3), probabilities are computed and provided in Table 10.

**Table 10:** Empirical probability distribution of entrepreneurs’ decisions against transforming sociocultural values following entrepreneurial attempts, controlling for geographical differences

“startplan”	“running”			“soon”			“only with favorable conditions “			“not to start”		
“place”|”msponge”	“negl”	“some”	“strong”	“negl”	“some”	“strong”	“negl”	“some”	“strong”	“negl”	“some”	“strong”
“north”	0.078	0.121	0.160	0.159	0.347	0.325	0.543	0.443	0.463	0.220	0.089	0.052
“south”	0.046	0.085	0.117	0.089	0.233	0.225	0.539	0.524	0.564	0.326	0.158	0.094
“central”	0.075	0.134	0.181	0.096	0.244	0.232	0.495	0.467	0.495	0.334	0.155	0.092

**Fig. 4:**
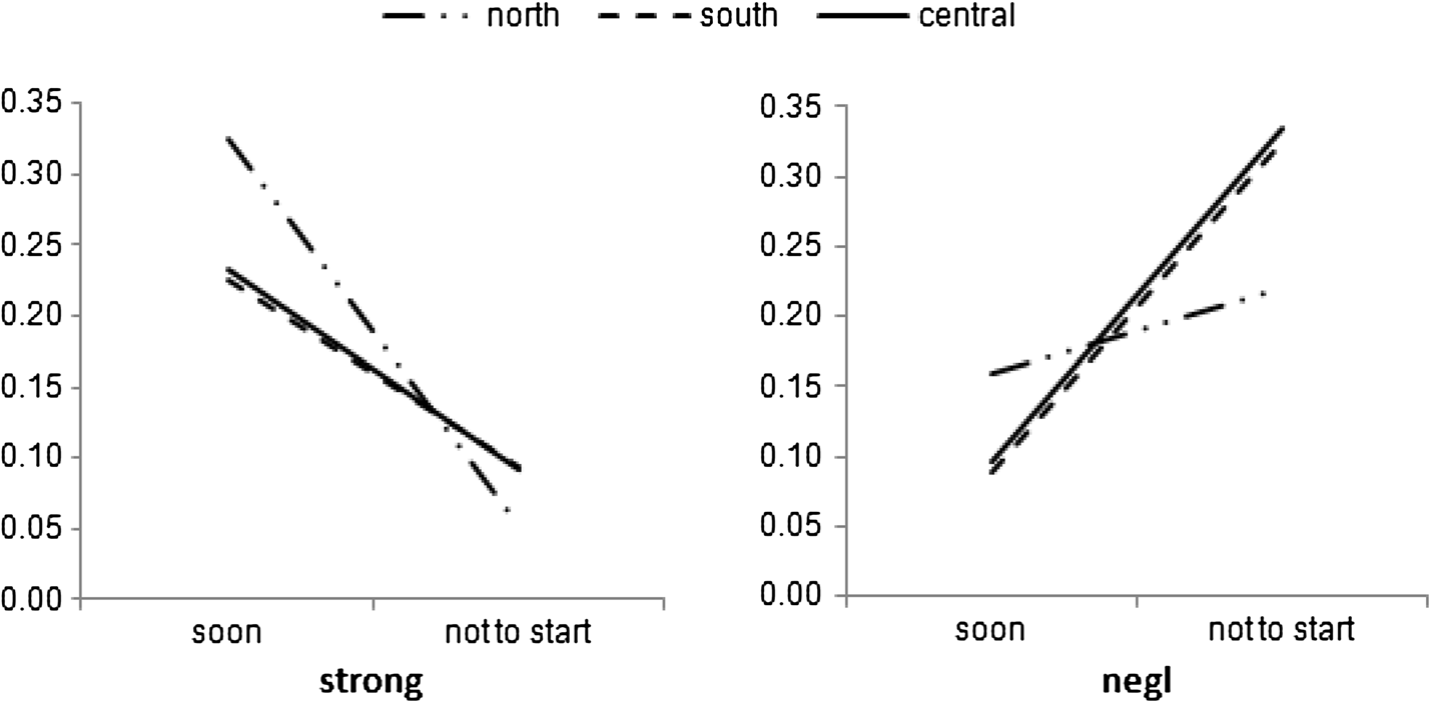
Outcomes of the mindsponge process effects help determine different trends of empirical probabilities for the entrepreneurial decision to start “soon” or “not to start”

A visual presentation of a subset of these probabilities, controlling for two values of mindsponge process outcome “strong” and “negl” is provided in Fig. 4.

In general, when the effect of the mindsponge process is strong, the probabilities decrease from starting soon to not starting; and the trend is in reverse when the mindsponge outcome is weak (negligible; “negl”). It is evident that although the trend is shared among entrepreneur groups in all three regions North, South, and Central, the North group shows a clear difference in the magnitudes of change as seen in Fig. 4.

### Estimations and results for RQ4

This last estimating effort is focused on the relationships between such factors as lessons learned from other entrepreneurial failures (“failurel”) and perceived likelihood of survival/success (“chance”), where “chance” serves as a group of response variables, and “failurel” predictors, using the same control variate of the geographical differences. Estimated results are provided in Table 11.

**Table 11:** Reported results from RQ4 estimations

	Intercept	“place”		“failurel”	
		“central”	“north”	“much”	“some”
	*β*_0_	*β*_1_	*β*_2_	*β*_3_	*β*_4_
logit(high|med)	−0.545** [−3.144]	0.296** [3.166]	0.456*** [3.627]	0.358^-,·^ [1.908]	−0.229 [−1.295]
logit(low|med)	−0.716*** [−3.802]	0.215^-,·^ [1.689]	0.264 [1.505]	−0.836*** [−3.745]	−0.801*** [−4.137]

Signif. codes: 0 ‘***’ 0.001 ‘**’ 0.01 ‘*’ 0.05 ‘·’ 0.1 ‘-’ 1, with z-value in square brackets. Baseline category for: “place”: “south”; and, “failurel”: “none”. Residual deviance: 8.426 on 8 d.f

The estimations yield significant relationships for variables entering the model, suggesting that learning from past failures would likely impact on perceived chance of success for entrepreneurs, from all three regions. Using the reported coefficients, Eqs. (RQ4.1–RQ4.2) represent relationships established through the empirical data set.

RQ4.1 $$\ln \left( {\frac{{\pi_{\text{high}} }}{{\pi_{\text{med}} }}} \right) = - 0.545 + 0.296{\text{Central}} + 0.456{\text{North}} + 0.358{\text{muchFail}} - 0.229{\text{someFail}}$$

RQ4.2 $$\ln \left( {\frac{{\pi_{\text{low}} }}{{\pi_{\text{med}} }}} \right) = - 0.716 + 0.215{\text{Central}} + 0.264{\text{North}} - 0.836{\text{muchFail}} - 0.801{\text{someFail}}$$

Likewise, Eqs. (RQ4.1–RQ4.2) allow the conditional empirical probabilities to be computed as provided in Table 12.

**Table 12:** Probability distributions of entrepreneurs’ perceived chance of success following learning from past failures, with control variate of geographical locations

“chance”	“high”			“med”			“low”		
“place”|”failurel”	“much”	“some”	“none”	“much”	“some”	“none”	“much”	“some”	“none”
“central”	0.469	0.328	0.327	0.421	0.528	0.419	0.110	0.144	0.254
“north”	0.506	0.361	0.359	0.387	0.497	0.392	0.107	0.142	0.249
“south”	0.406	0.274	0.280	0.490	0.595	0.484	0.104	0.131	0.236

Table 12 shows that the perceived chance of success improves when entrepreneurs spend time to study past failures of other entrepreneurial attempts, and vice versa.

## Conclusion

The above analysis has yielded confirmatory effects of such factors as active participation in social networks, CSR willingness, transformed sociocultural values and lessons from past failures on determining: i) entrepreneurial creativity (RQ1); perseverance (RQ2); decisiveness (RQ3); and, perceived likelihood of success (RQ4), together with empirically established relationships among them and computed empirical probabilities, controlling for geographical differences. This final section offers a brief discussion with remarks on useful insights.

### Networked creativity

Figure 1 and the empirical computations confirm the so-called “networked creativity” phenomenon suggested by Harryson (2008). The known impact of geographical location also suggests that the North group of networked entrepreneurs tend to report a higher reliance on creative performance in their entrepreneurial attempts, followed by the South group, then the Central group.

The “networked creativity” is also profound when taking geographical locations into account as for strongly networked entrepreneurs, the trends of probability change vary, with a similar trend but of varying magnitudes for the South and Central groups, but an opposite trend for the North group.

The result is important as up until now, sociocultural differences among Vietnamese businesspeople have been described in qualitative terms, and are generic, making it difficult to predict management outcomes and associated conditions. Place has a verified influence on outcomes of creative performance, most probably because entrepreneurial behaviors have been substantially influenced by such habits as use of time, funds, savings and self-motivations in achieving business goals.

### CSR-supported perseverance

There appears to have been a close relationship between one’s unwillingness to take part in CSR activities and one’s lower degree of perseverance (<24 months) in the entrepreneurial attempt. This perhaps reflects a noteworthy transforming of sociocultural traits of perseverant entrepreneurs: CSR-willingness. It is highly possible that because of the willingness and belief in social values of CSR, an entrepreneur may have higher trust in their final success, and the reverse also holds.

Entrepreneurs from the central region appear to be more CSR-willing, with a probability of 80.6 % for the subgroup of CSR-willing from the Central group to remain perseverant in their startup endeavors. In Vietnam, people from the Central region are often regarded as hard working, socially supportive, and showing humility. Figure 2 also shows that the “perseverance probability line” of the Central group is above that of the North and South groups (left-hand side figure).

Again the outcome of this examination shows that “social embedding” can somehow serve as an ideal for maintaining entrepreneurs’ perseverance, especially over tumultuous periods of transition. This understanding is non-trivial as the term “transitional economies” tend to be perceived as “homogeneous” while the reality shows that the term has even been changing within one economy, over a relatively short period of time. And this is most probably not country-specific; and the evidence found in Vietnam may someday be directly comparable to evidence found in other emerging countries. In addition, geographical locations influence the degree of difference among groups of entrepreneurs. In our observation, these differences are directly related to how local societies and organizations are structured, together with power hierarchy associated with them.

### Mindsponge-based decisiveness

The computations of empirical probability support the theoretical value of the “mindsponge” concept as developed in Vuong and Napier (2015), with all coefficients being statitically significant. More importantly, when its value is “negligible” (that is, the entrepreneurs’ mindsponge outcome is minimal or virtually non-existent) the probabilities of entrepreneurs’ decisiveness in their business action diminish. In contrast, when its value is “strong,” entrepreneurs’ decisiveness increases sharply (see Fig. 3). Apparently, these do not happen by chance but due to entrepreneurs’ capabilities of transforming sociocultural values to match with their plans and pursuits, when taking up their role as an entrepreneur. In addition, since mindsponge deals with the transforming of beliefs and values, different geographical regions should theoretically show varying degrees of effect. Both Fig. 3 and probabilities reported in Table 10 are useful in verifying this hypothetical statement. There is evidence that the distinct difference is seen with the North group, while the remaining groups show a similar trend. This helps to partly explain why there are more Northern business people starting in the Central and South regions than the other way around. In general, the stronger the mindsponge process is, the more willing and decisive the entrepreneur appears to become. It may suggest that the demand for transforming sociocultural values within the entrepreneur community is higher than the Vietnamese society normally thinks about.

This new result suggests that Vietnamese entrepreneurship appears to have become increasingly receptive to environmental changes; and a more politically and economically complex structure of the North enables entrepreneurs to see the changing process as more far-reaching and flexible than their counterparts in the Central and South regions.

### Improved chance of success based on lessons from the past

Although the empirical observations and results establish that learning from past failures helps improve the confidence of entrepreneurs, the limit is still 51 % for the high chance of success group. This empirical probability is almost the same as tossing a coin (see Table 12). Its main meaning, as expected, confirms the effect of decreasing the probabilities of low perceived chance (<50 %).

**Fig. 5:**
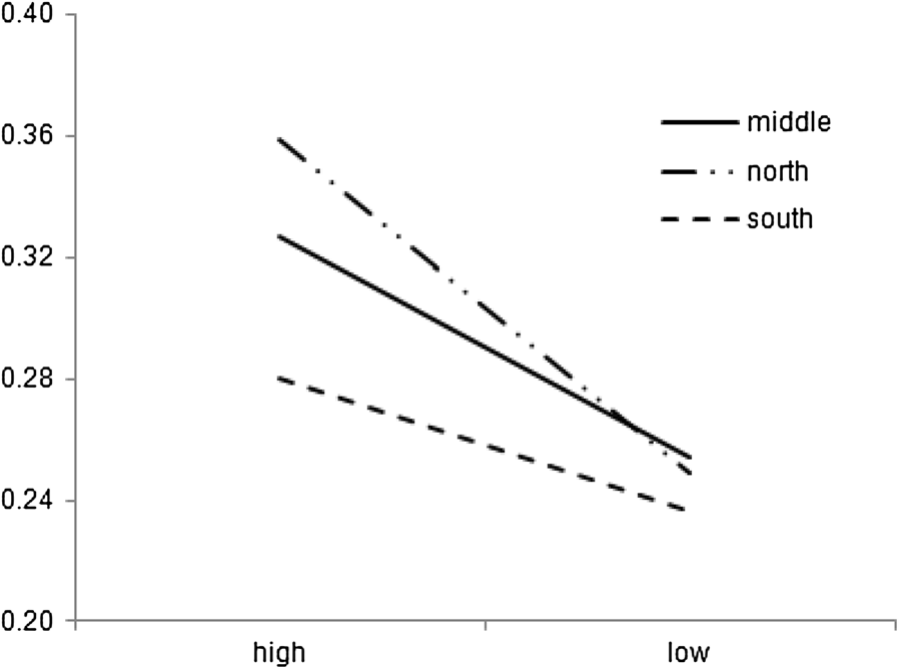
Probabilities of chance for those without knowledge about others’ past failures

In addition, Fig. 5 shows estimated probabilities of chance of success for those without knowledge about others’ past failures, controlling for geographical differences; and it is noteworthy. Entrepreneurs who have no knowledge about others’ failure lessons tend to agree on a probability of low chance but disagree on the probabilities of higher chance of success. More strikingly, for those without knowledge of past failures, they tend to overestimate their chance of success, regardless of where they come from. The most overoptimistic are Northern entrepreneurs.
